# Degos disease: a case report and review of the literature

**DOI:** 10.1186/s13256-020-02514-6

**Published:** 2020-10-29

**Authors:** Santosh Tummidi, Prabhakaran Nagendran, Swaroopa Gedela, Jami Rupa Ramani, Arundhathi Shankaralingappa

**Affiliations:** 1 Department of Pathology, All India Institute of Medical Sciences, Mangalagiri, Andhra Pradesh India; 2 Department of Dermatology, All India Institute of Medical Sciences, Mangalagiri, Andhra Pradesh India

**Keywords:** Degos disease, Malignant atrophic papulosis, Skin biopsy, Vasculopathy

## Abstract

**Background:**

Degos disease is a very rare syndrome with multisystem vasculopathy of unknown cause. It can affect the skin, gastrointestinal tract, and central nervous system. However, other organs such as the kidney, lungs, pleura, and liver can also be involved.

**Case presentation:**

A 35-year-old Hindu woman presented to our dermatology outpatient department with complaints of depigmented painful lesions. A skin punch biopsy taken from the porcelain white atrophic papules which revealed features of Degos disease.

**Conclusion:**

The diagnosis of Degos disease is usually based on the presence of the pathognomonic skin lesions and a tissue biopsy demonstrating a wedge-shaped area of necrosis with thrombotic occlusion of the small arterioles. No specific treatment is currently available for this disease.

## Background

Degos disease (DD), also called “malignant atrophic papulosis” or “lethal cutaneous and gastrointestinal arteriolar thrombosis,” is a rare occlusive arteriopathy having unknown pathogenesis and involves predominantly small-caliber vessels of the dermis, gastrointestinal tract, central nervous system, and occasionally other organs [1–3]. Not more than 200 cases are reported in the literature [1, 4].

## Case presentation

**Fig. 1:**
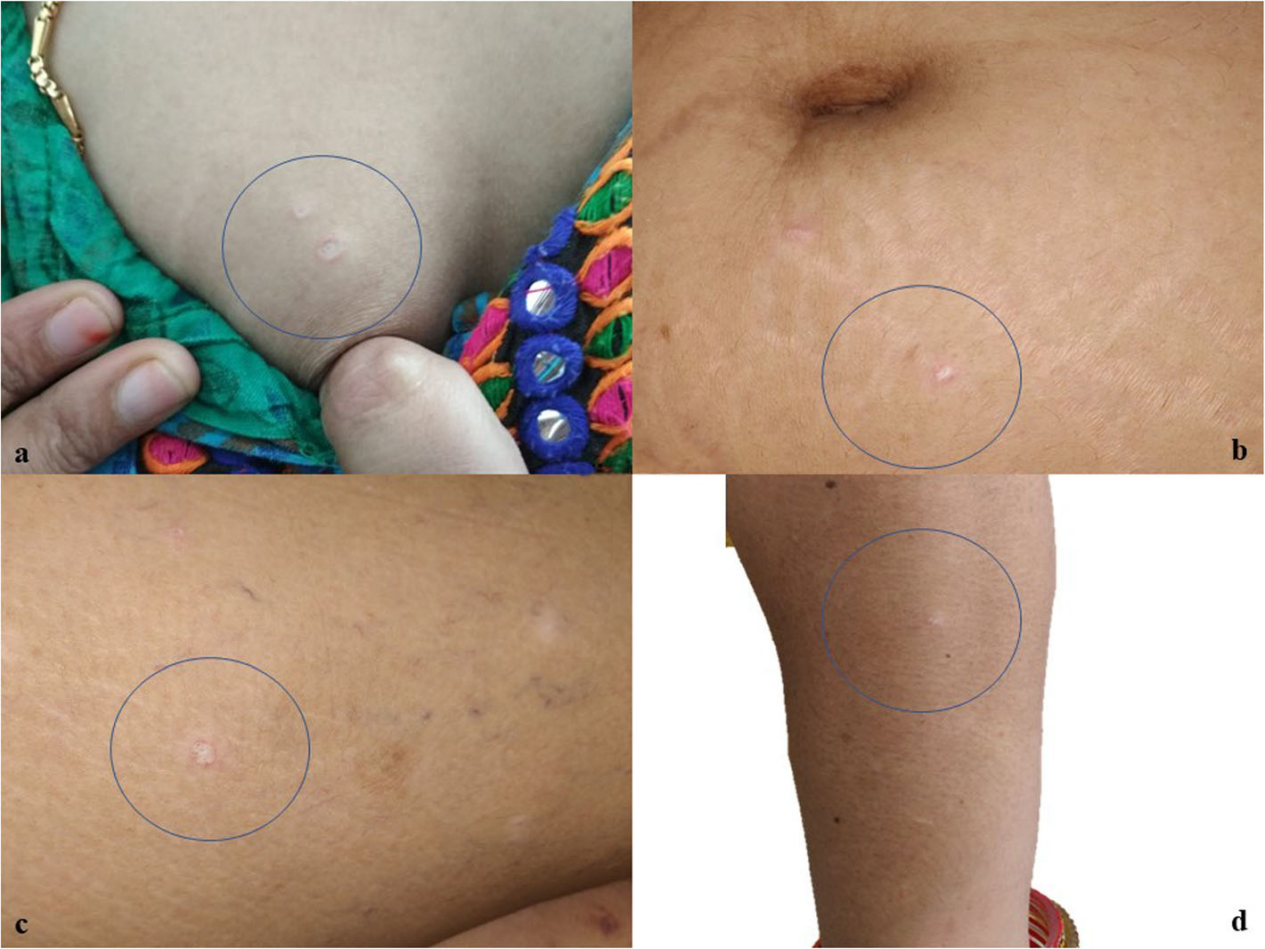
Porcelain white atrophic papules with surrounding erythema present over chest (**a**), periumbilical area (**b**), thigh (**c**), and right upper limbs (**d**)

A 35-year-old Hindu woman presented to our dermatology outpatient department with complaints of painful depigmented lesions of 1½ years’ duration. On examination, the lesions were porcelain white atrophic papules with surrounding erythema present over the trunk, upper limbs, thighs, and chest (Figure 1a-d). The lesions were associated with mild tenderness. She had a history of joint pain for the last 7–8 years and a history of headache, giddiness, and ocular pain for the last 3 months. She had no history of proximal muscle weakness or skin tightening. Her menstrual cycles were regular. She had a history of irregular bowel habits (constipation). She had no history of hypertension or diabetes/any other medication. Results of her laboratory tests for antinuclear antibody, lupus anticoagulant, cardiolipin antibody, and β_2_-glycoprotein were all negative. Ultrasonography of the abdomen was unremarkable. Ophthalmological examination of bilateral eyes showed myopic astigmatism. The result of stool occult testing of the patient’s blood was negative.

**Fig. 2:**
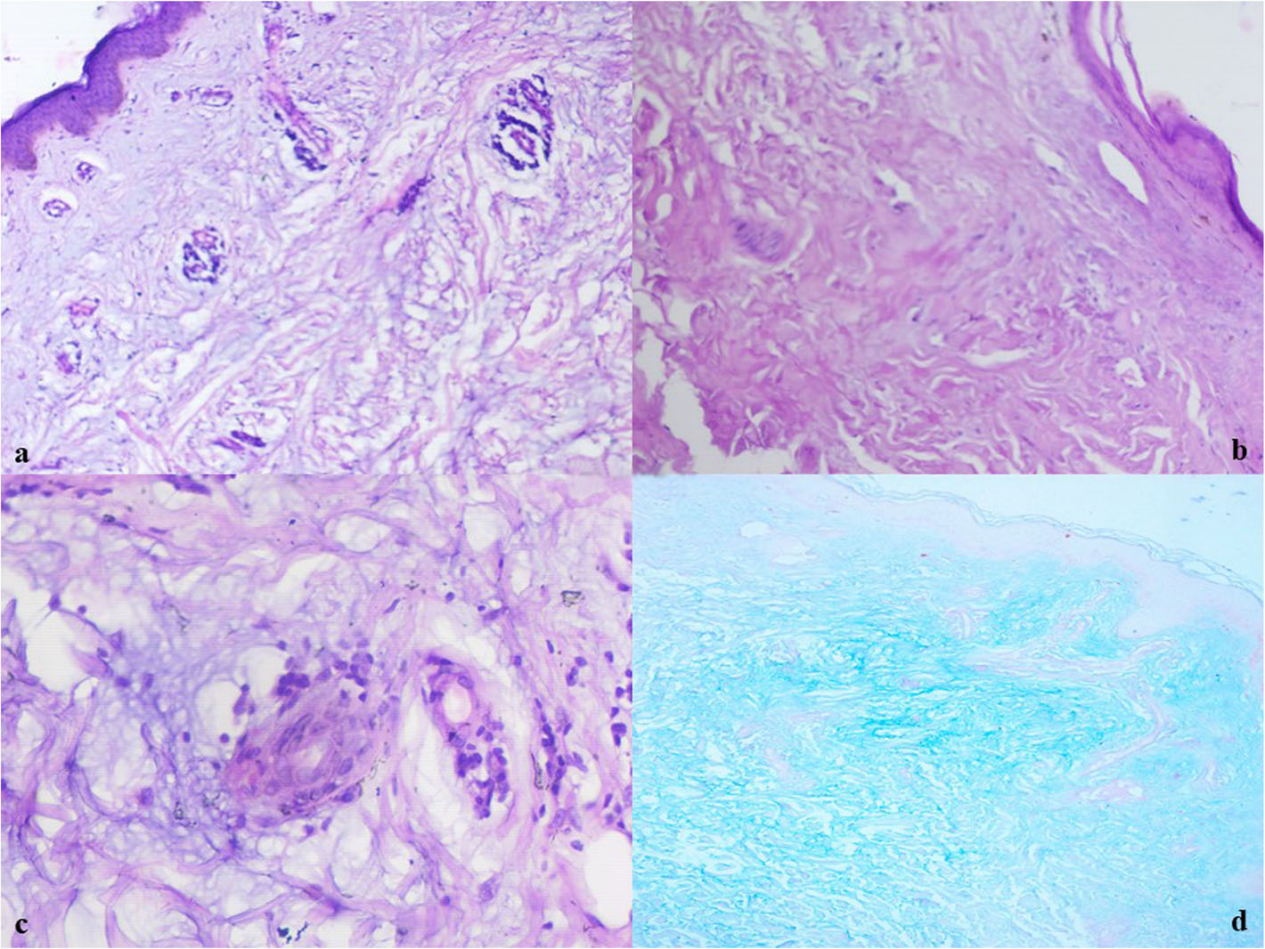
**a** Microscopy shows atrophic epidermis with flattened rete ridges. Papillary dermis is showing band-like mucin deposit along with focal sclerosis. **b** Focal wedge-shaped fibrosis is noted in the middle and deep dermis with patchy mucin. **c** Mild cuffing of lymphomononuclear infiltrate around perivascular areas in the dermis. **d** Alcian blue staining for dermal mucin is positive (Hematoxylin and eosin stain (**a**-**c**); original magnifications × 10, × 40; Alcian blue stain (**d**); original magnification, × 10)

A skin punch biopsy was taken from the porcelain white lesion over the trunk. Histopathological examination of the sections showed atrophic epidermis with mild hyperkeratosis and flattened rete ridges focally. The underlying papillary was showing a band-like mucin deposit along with focal sclerosis. The middle and deep dermis had patchy mucin along with focal fibrosis. Cuffing of mild to moderate lymphomononuclear infiltrate was observed around perivascular areas in the dermis. Few of the blood vessels were showing endothelial cell proliferation (Figure 2a-c). Scant subcutaneous tissue was available in the biopsy. The result of Alcian blue staining for dermal mucin was positive (Fig. 2d). Owing to the absence of systemic complications, the patient was diagnosed with benign cutaneous Degos disease.

**Fig. 3:**
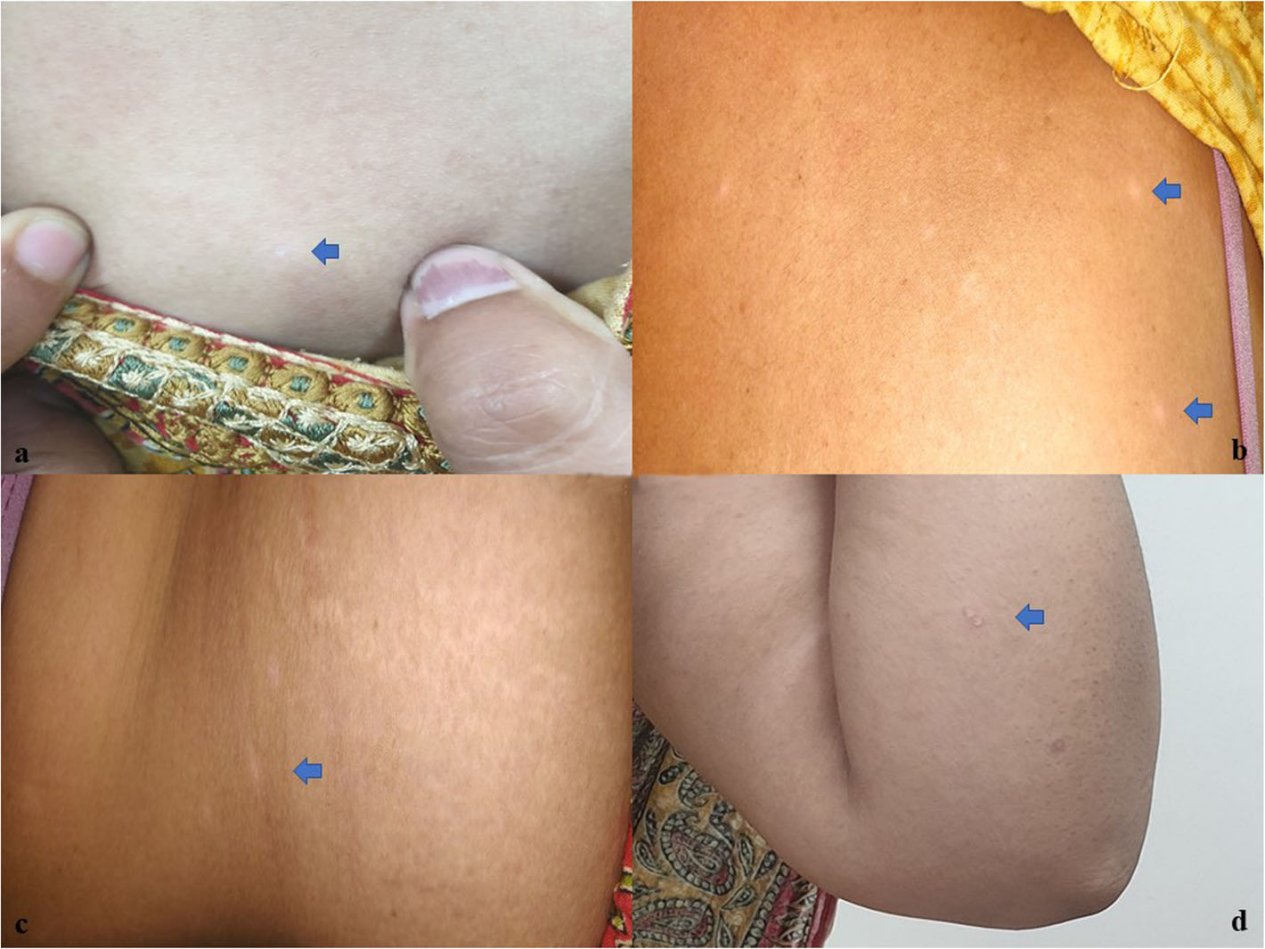
Post-treatment pictures revealing improvement of lesions in the chest (**a**), anterior abdominal wall (**b**), trunk (**c**), and right arm (**d**)

 She was prescribed antiplatelet agents (aspirin 75 mg and clopidogrel 75 mg once daily). New lesions stopped appearing within 1 month of starting this therapy. Patient’s follow-up after 3 months, the lesions had started flattening, the erythema had decreased, and her pain was completely subsided (Figure 3a-d). The patient is under regular follow-up with us, and her condition is improving.

## Discussion and conclusion

Kohlmeier first described DD in 1941 [2, 5]. The following year, Degos presented DD as a distinct entity and coined its name [3]. DD, also known as “malignant atrophic papulosis,” is a rare systemic vaso-occlusive disorder with unknown pathophysiology [6]. Some authors have stated that DD might result from coagulopathy, vasculitis, or endothelial cell dysfunction [5, 7]. Anticoagulation strategies targeted to coagulopathy and immunosuppressive modalities that were successful with vasculitis have proved to be inadequate in treating systemic disease [7]. The characteristic raised papules with a red border and a depressed “china white” center are possibly due to dermal capillaries and venules developing a thrombogenic microangiopathy [5].

DD is more common and severe in males than in females. Most cases are sporadic, although a familial variant with an autosomal dominant pattern has also been described [1]. The first manifestation of DD is skin rash, and in about 15% of patients, the disease remains limited to skin (benign form), whereas in others, it progresses to systemic involvement (malignant form and universally fatal) [8].

The prognosis of DD depends on systemic involvement. The cutaneous benign form may persist for years without the involvement of the internal organs. The malignant form includes simultaneous or subsequent involvement of the internal organs (for example, multiple limited infarcts of the intestines, central nervous system, lungs [pleuritis and/or pericarditis], and eyes), leading to a 50% risk of death within 2 to 3 years after symptoms appear. Bowel perforation resulting in peritonitis is a common cause of death in patients with malignant atrophic papulosis [9].

An increased expression of both MxA (type I interferon–inducible protein) and complement C5b-9 (membrane attack complex) in the endothelial cells, vessel walls, perivascular interstitium, inflammatory cells, and keratinocytes has been demonstrated, suggesting that complement-mediated injury to endothelial cells may be involved in the pathogenesis [9, 10].

The diagnosis of malignant atrophic papulosis is usually based on the presence of pathognomonic skin lesions and a tissue biopsy demonstrating a wedge-shaped area of wedge necrosis with end arterial thrombotic occlusion of the small arteries and infraction of dermis [1, 6, 9, 11].

The differential diagnosis includes primary antiphospholipid syndrome or the antiphospholipid syndrome caused by systemic lupus erythematous or other connective tissue diseases. Malignant atrophic papulosis is a vaso-occlusive disorder of unknown cause [9, 12].

To date, there have been no clear guidelines for the treatment of DD. Antiplatelet agents such as aspirin, dipyridamole, and clopidogrel have been found to be effective [11]. Acutely ill patients have been treated with heparin with success. However, other fibrinolytic agents were ineffective [5]. Immunosuppression with corticosteroids has been shown to worsen skin lesions and further complicate the course of the disease [5]. The efficacy of eculizumab (monoclonal antibody targeted against complement C5) and treprostinil (synthetic prostaglandin agonist) has been reported in the literature [9, 12]. For gastrointestinal tract perforation, surgical intervention is the only choice however recurrent perforations may still develop [9, 12]. Systemic manifestations might develop suddenly or even years after the occurrence of skin lesions, indicating the need for annual follow-up [11].

DD is a rare, chronic, occlusive vasculopathic disease. There is no specific laboratory test that can help in the diagnosis of this disease. Gastrointestinal involvement can cause serious and lethal disease. Pathognomonic skin lesions and clinical suspicion with punch biopsy helped in the correct diagnosis of our patient. The follow-up strategy includes clinical examination of the cutaneous lesions with additional systemic monitoring to assess long-term prognosis.

## Data Availability

All the data regarding the findings are available within this report.

## Abbreviation

DD: Degos disease
